# Supplementary material for: Genetic footprint of population fragmentation and contemporary collapse in a freshwater cetacean
Source: Sci Rep. 2017 Oct 31;7:14449. doi: 10.1038/s41598-017-14812-z (PMC5663847; doi:10.1038/s41598-017-14812-z)
Supplement: Supplementary file 1 — Supplementary information [file 41598_2017_14812_MOESM1_ESM.pdf]

Supplementary Materials

Genetic footprint of population fragmentation and contemporary collapse in a freshwater cetacean

Minmin Chen[†], Michael C. Fontaine[†*], Yacine Ben Chehida[†], Jinsong Zheng[*], Frédéric Labbé, Zhigang Mei, Yujiang Hao, Kexiong Wang, Min Wu, Qingzhong Zhao, Ding Wang[*]

Content

Appendix S1 | Supplementary details on the ABC analyses.

Figure S1 | Estimated probability of the data (X) given K group tested in *Structure*.

Figure S2 | Scatter plot showing the individual scores along the first two principal components of the Principal Component Analysis.

Figure S3 | Allelic richness estimated using ADZE for each group identified in *Structure*.

Figure S4 | Flow chart about inference of population history using ABC in the program DIYABC.

Figure S5 | Performance of the ABC-RF in selecting the best model (among ten) of the step (*a*) in Fig. 4a.

Figure S6 | Performance of the ABC-RF in selecting the best model (among six) of the step (*bi*) in Fig. 4b.

Figure S7 | Performance of the ABC-RF in selecting the best model (among two) of the step (*bii*) in Fig. 4b.

Table. S1. Estimated recent migration (upper and lower matrix) and non-migration rates (along the diagonal) per generation between populations identified by *STRUCTURE*

Table S2 | Model specification and prior distributions for demographic parameters.
See figure 2 for the demographic parameters of each model tested.

Table S3 | Mutation model parameters for the ABC analysis figure 4.

Table S4 | Model selection procedure and performance analysis for the ABC step 1.

Table S5 | Model checking for the first step.

Table S6 | Model selection procedure and performance analysis for the ABC step (*bi*), figure 4*b*.

Table S7 | Model checking for the ABC step *bi* (figure 4*bi*).

Table S8 | Model selection procedure and performance analysis for the ABC step (*bii*), figure 4*b*.

Table S9. Model checking for the analysis for the ABC step (*bii*), figure 4*b*.

Table S10 | Parameter estimation for scenario SC2 in natural units (figure. 4b_{ii}).

Table S11 | Composite parameter for the scenario SC2 (see figure 4*bii*).

---

† These authors contributed equally and should be considered as co-first authors.

* Corresponding authors: Michael C Fontaine (mikafontaine@gmail.com); Ding Wang (wangd@ihb.ac.cn); Jinsong Zheng (zhengjinsong@ihb.ac.cn)

Appendix S1. Supplementary details on the ABC analyses

Model parameters. The parameters defining each scenario (*i.e.*, effective population sizes *Ne*, times of population size changes and population splits *T*, and mutation rates μ) were considered as random variables drawn from prior distributions (figure 4, table S3, S4). For each simulation, DIYABC draw a value for each parameter from its prior distribution and performed coalescent simulations to generate a simulated pseudo-observed dataset or POD with the same number of gene copies and loci per population as in the observed dataset.

Coalescent simulations assume a mutation model for each type of loci. The mutation model for microsatellite loci was a generalized stepwise-mutation (GSM) model [1] with two parameters: a mean mutation rate ($\bar{\mu}_{mic}$) and mean of the geometric distribution for the length, in repeat numbers, of mutation events ($\bar{P}$) drawn from uniform prior distributions ($\bar{\mu}_{mic}$: [10^{-3} – 10^{-4}] and $\bar{P}$: [0.1-0.3], see Table S4). We accounted for variation in μ_{mic} and *P* among loci by drawing their individual values from a gamma distribution (Table S4). These settings allowed for large mutation rate variance across loci (i.e. range of 10^{-5} to 10^{-2}). We also considered mutations inserting or deleting a single nucleotide in the microsatellite sequence.

We used jModelTest 2.1.7 [2] to identify the best substitution model describing the sequence variation of the mtDNA-CR and estimate its parameters. The best mutation model was a HKY+I+G model [3] with a proportion of constant sites of 16%, and a shape of the gamma distribution of mutations among sites equal to 99.8 (Table S4). We assumed a per-site and per-generation mutation rate ranging uniformly between 1×10^{-7} and 1×10^{-5}, as found in the literature [4,5].

Model selection procedure using the ABC-Linear Discriminant Analysis (LDA) procedure. For each step in the ABC analysis (Fig. 4), we simulated 1×10^6 PODs for each scenario tested using DIYABC. A set of 48 summary statistics (SS) describing within and among population genetic diversity were calculated with DIYABC for each POD and the observed data. Within population statistics for microsatellite loci included the mean number of alleles per locus (NAL), expected heterozygosity (HET), allele size variance (VAR), M_{GW} statistic of Garza & Williamson across loci [6]. Between population statistics for microsatellites included F_{ST} [7], shared allele distance (DAS) [8], and $(\delta\mu)^2$ Goldstein's distance [6,9]. For the mtDNA data, the descriptive statistics within populations include the number of haplotype (NAH), the number of segregating sites (NSS), the mean pairwise differences (MPD) and its variance (VPD), Tajima's *D*, and the number of private segregating sites (PSS). Statistics computed between groups were the mean of within sample pairwise differences (MP2), mean of between sample pairwise differences (MPB), and H_{ST} between two samples [7,10]. A Euclidean distance was calculated between the statistics obtained for each normalized PODs and observed dataset [8,11].

We used the standard ABC-LDA procedure to estimate the Posterior probability (PPr) of each competing scenario using a polychotomous logistic regression [12,13] on the 1% of simulated datasets closest to the observed dataset (lowest Euclidean distance δ), subject to a linear discriminant analysis on the summary statistics as a pre-processing step (to reduce the dimensionality of the data) [14] and avoid the "curse of dimensionality" [15,16]. The best-fitting scenario was selected based on the highest PPr value with a non-overlapping 95% confidence interval (95% CI).

Model selection procedure using the ABC- Random Forest (RF). Both theoretical arguments and simulation experiments indicate that approximate PPr estimated from standard ABC analyses for the modeled demographic scenarios can be inaccurate, even though the models being compared can still be ranked appropriately using numerical approximation [17]. To over-come this problem, we complemented the standard ABC-LDA model choice procedure with the newly developed approach based on a machine learning tool named "random forests" (ABC-RF), which selects among the complex introduction models covered by ABC algorithms [15]. The ABC-RF analysis provides a classification vote representing the number of times a scenario is selected as the best one among *n* trees in the constructed random forest. The scenario with the highest number of classification vote was selected as the best scenario among a total of 500 classification trees [15]. Posterior probabilities and prior error rates (i.e. the probability of choosing a wrong model when drawing model index and parameter values into the priors of the best scenario) were computed over 10 replicate analyses, as suggested in [18]. We used the *abcrf* v.1.5.0 R statistical package [15] to conduct the ABC-RF analyses on a reference table that includes 1×10^4 PODs from which were calculated a total of 114 summary statistics (SS). They include all the SS used in the ABC-LDA analyses augmented with the Mean index of classification (relationship between two samples) [19,20] and the linear discriminant functions (LDA) as additional synthetic variables, following the recommendation of [15,18].

Performance of the model choice procedure. We evaluated the ability of the ABC analysis to discriminate between the competing scenarios by estimating the prior error rate under the ABC-RF [15]. We also use the more standard approach by analyzing 300 simulated data sets with the same number of loci and individuals as our real data set under the ABC-LDA. Following the standard ABC-LDA procedure [13], we estimated the Type-I error rate as the proportion of instances in which the selected scenario did not show the highest posterior probability among the competing scenarios, for the 300 simulated datasets generated under the best-supported scenario. Similarly, we estimated the Type-II error rate, by simulating 300 data sets for each of the other competing scenarios and calculating the mean proportion of instances in which the best-supported model was incorrectly selected as the most probable scenario.

Estimation of marginal posterior distribution of the model parameters. We estimated the posterior distributions of each demographic parameter under the best demographic model, by carrying out local linear regressions on the 1% closest of 10^6 simulated data sets, after a logit transformation to parameter values [11,12].

Goodness-of fit of the fitted model to the data. Finally we conducted a model checking procedure implemented in DIYABC to evaluate the goodness-of-fit between the posterior parameter distribution and the observed data following [21]. For this analysis, we simulated 1,000 pseudo-observed datasets under each model-posterior combination, with sets of parameter values drawn with replacement from the parameter posterior distribution. This generated a posterior cumulative distribution function for each summary statistic allowing us to estimate how well each fitted model can reproduce the observed summary statistics.

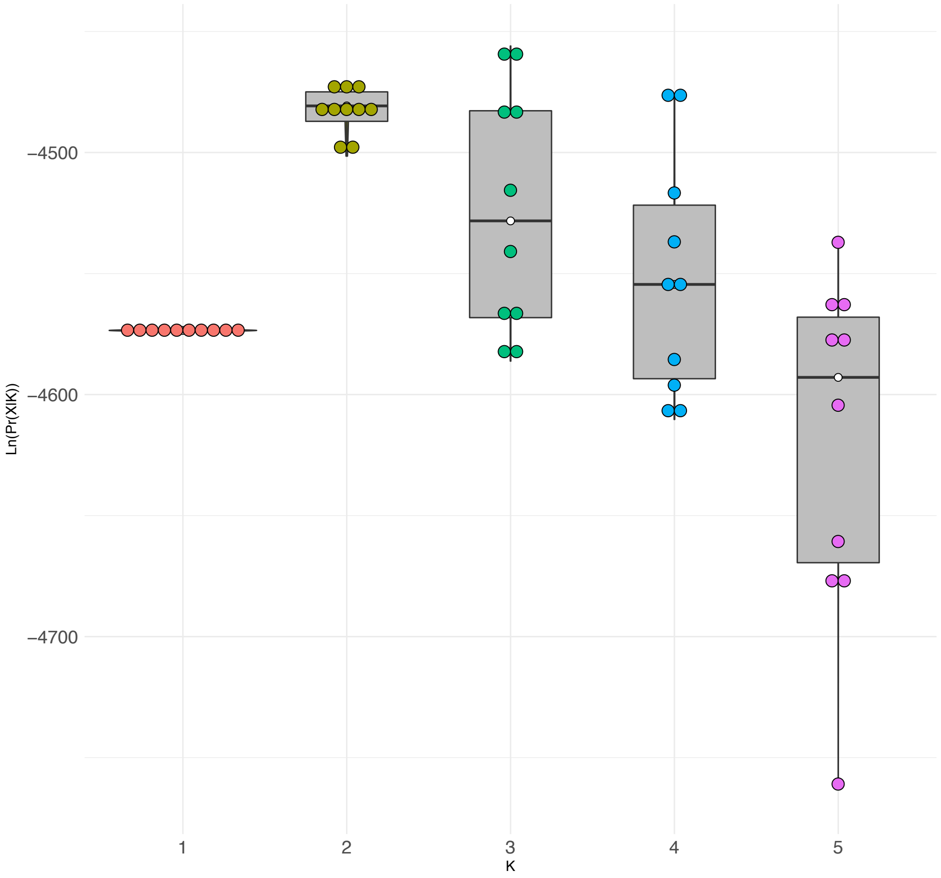

Figure S1 | Estimated probability of the data (X) given K group tested in *Structure*.

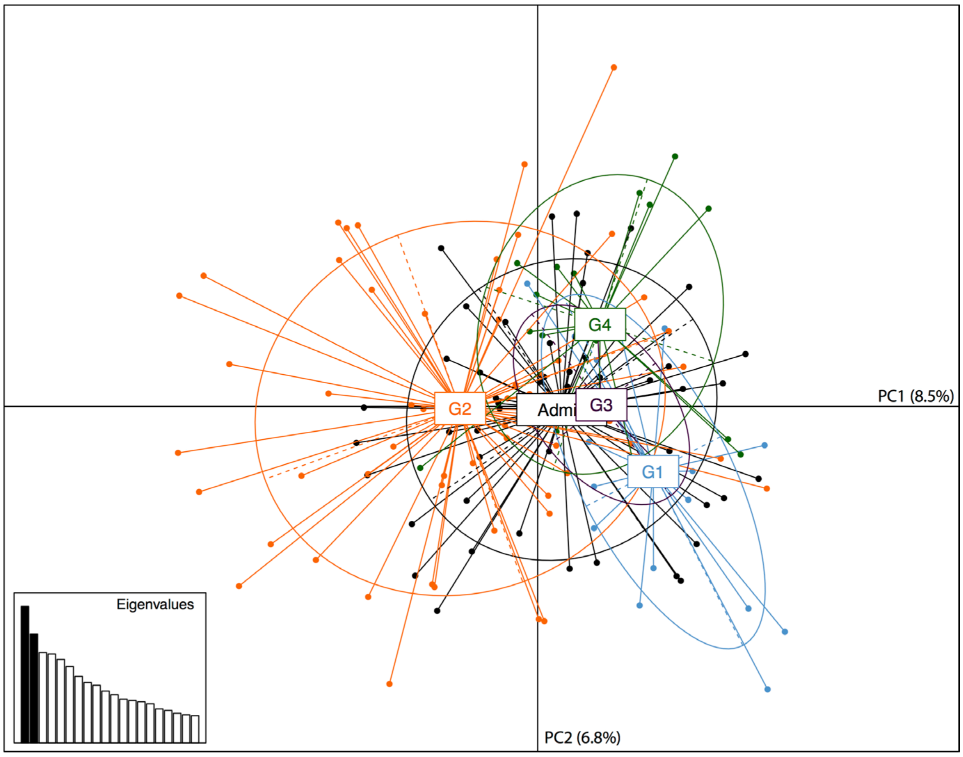

Figure S2 | Scatter plot showing the individual scores along the first two principal components of the Principal Component Analysis. The proportion of variance explained by each axis and the firsts eigenvalues (inset) are provided.

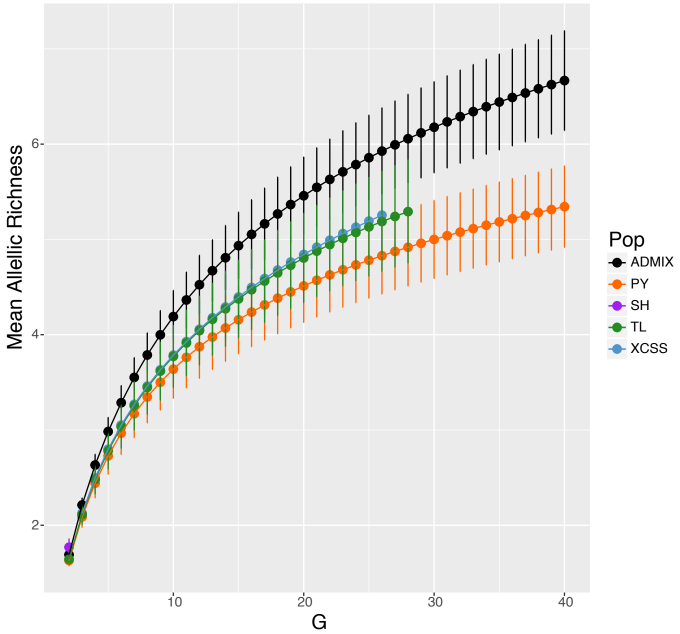

Figure S3 | Allelic richness estimated using ADZE [22] for each group identified in *Structure*. Variation in *Ar* and *pA* are provided as a function of the sample size considered in the rarefaction procedure.

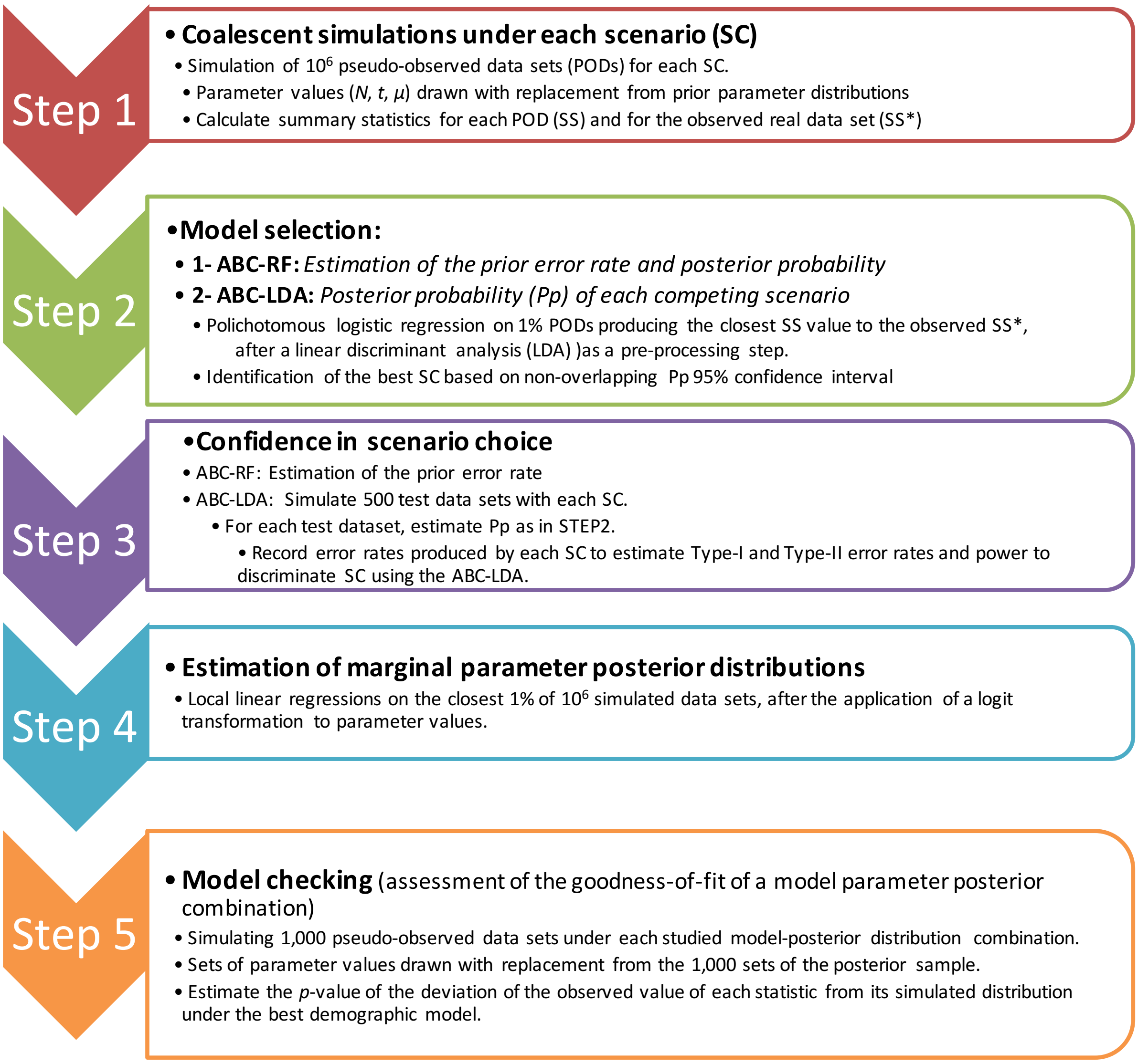

Figure S4 | Flow chart about inference of population history using ABC in the program DIYABC.

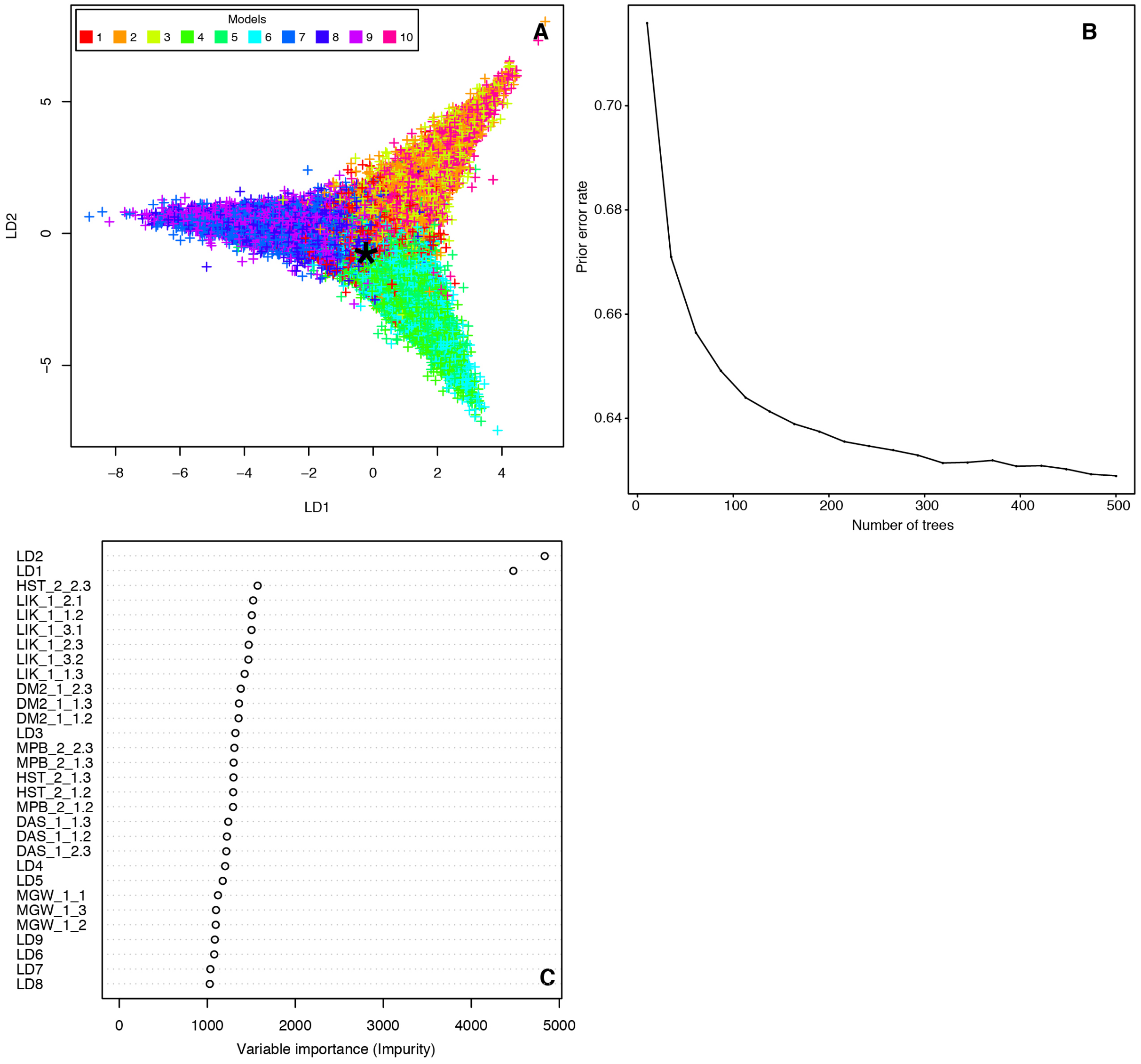

Figure S5 | Performance of the ABC-RF in selecting the best model (among ten) of the step (*a*) in Fig. 4a. (A) Projection of the reference table on the first two Linear Discriminant Analysis (LDA) axes. Colors correspond to model indices (*Fig. 4a*). The location of the observed dataset is indicated by a large black star. (B) Evolution of the ABC-RF prior error rate with respect to the number of trees in the forest. (C) Contributions of the thirty most important statistics to the RF to discriminate among the ten scenarios. The contribution of a statistic is evaluated as the mean decrease in node impurity in the trees of the RF [15]. The meaning of the variable acronyms is provided in Appendix S1.

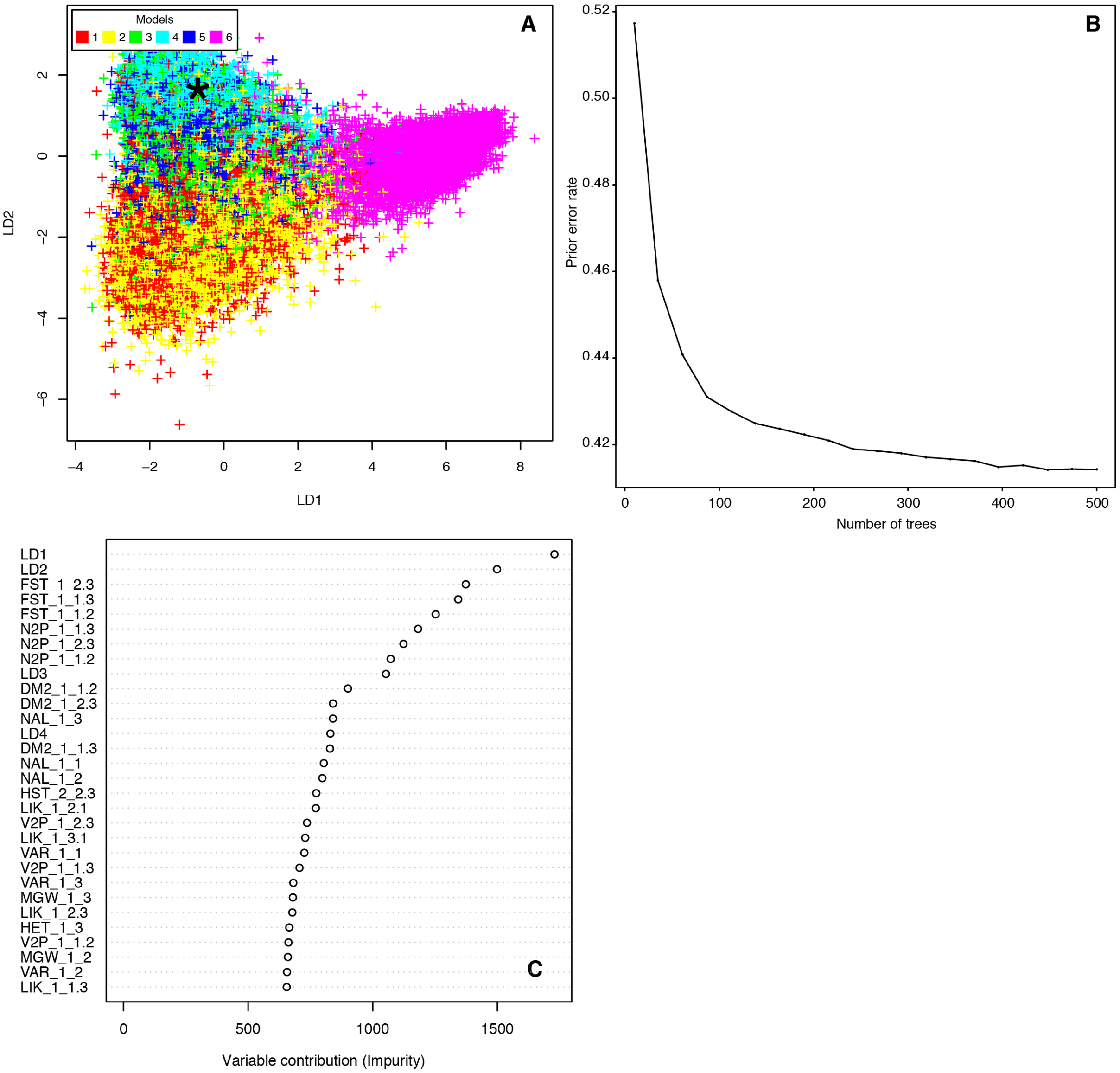

Figure S6 | Performance of the ABC-RF in selecting the best model (among six) of the step *bi* in Fig. 4b.
See Fig. S5 for the legend.

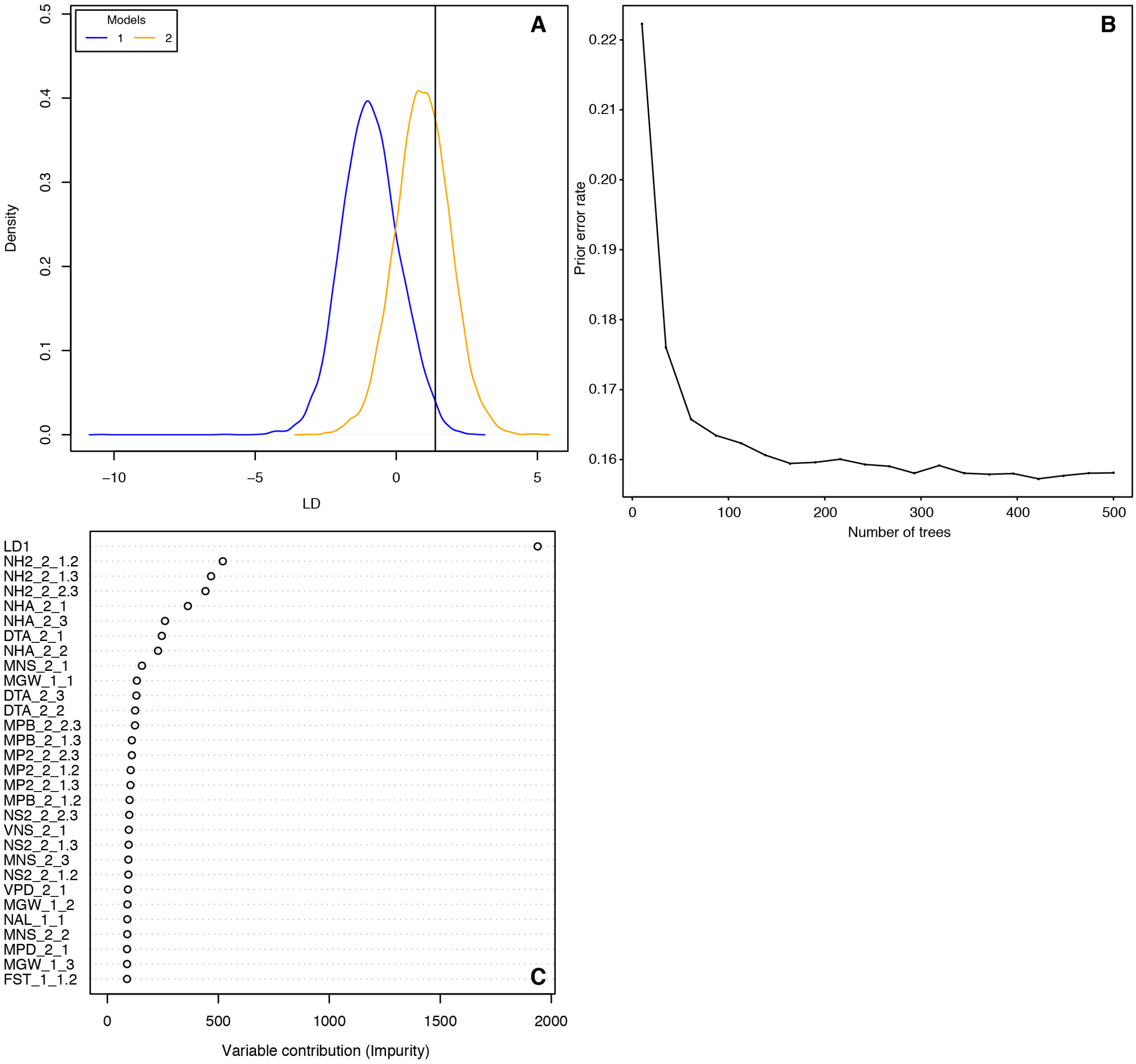

Figure S7 | Performance of the ABC-RF in selecting the best model (among two) of the step *bii* in Fig. 4b. See Fig. S5 for the legend.

Table. S1. Estimated recent migration (upper and lower matrix) and non-migration rates (along the diagonal) per generation between populations identified by *STRUCTURE*. Values are provided as geometric mean (median) [95% Highest probability density intervals].

To: / From:	**Admix**	**PY**	**TL**	**XCSS**
**Admix**	0.792 (0.791) [0.696 – 0.893]	0.011 (0.014) [0.000 – 0.045]	**0.208 (0.238) [0.085 – 0.315]**	**0.276 (0.282) [0.222 – 0.326)**
**PY**	**0.185 (0.195) [0.095 – 0.292]**	0.972 (0.975) [0.941 – 0.997]	0.056 (0.061) [0.000 – 0.211]	0.015 (0.019) [0.000 – 0.069]
**TL**	0.004 (0.005) [0.000 – 0.019]	0.003 (0.004) [0.000 – 0.016]	0.682 (0.678) [0.667 – 0.713]	0.009 (0.011) [0.000 – 0.045]
**XCSS**	0.004 (0.005) [0.000 – 0.022]	0.003 (0.004) [0.000 – 0.016]	0.009 (0.011) [0.000 – 0.046]	0.682 (0.678) [0.667 – 0.711]

(non-zero migration rate values are in bold font)

Table. S2: Model specification and prior distributions for demographic parameters. See figure 4 for the demographic parameters of each model tested.

Step a in Fig. 4

**Demographic Parameter**	**Type**	**Prior**
N_1	N	UN~[1–5,000]
N_2	N	UN~[1–5,000]
N_3	N	UN~[1–5,000]
N_a	N	UN~[10–20,000]
N_{T1}	N	UN~[10–10,000]
T_{rad}	T	UN~[10–5,000]
T_1 ($< T_2$)	T	UN~[10–10,000]
T_2	T	UN~[10–10,000]

Type of parameters: (*N*) effective population size, (*T*) time of the event in generation. Uniform distribution (UN) with 2 parameters: min and max; Gamma distribution (GA) with 4 parameters: min, max, mean and shape; Log-Uniform (LU) distribution with 2 parameters: min and max.

Table. S2 (*Continue)*

Step b_i in Fig. 4

Demographic Parameter	Type	Prior
N11	N	UN~[1-5000]
N21	N	UN~[1-5000]
N31	N	UN~[1-5000]
Nanc1	N	UN~[10-20000]
Tanc1 / Trad	T	UN~[10-5000]
N12 (>Nbot12)	N	UN~[1-5000]
N22 (>Nbot22)	N	UN~[1-5000]
N32 (>Nbot32)	N	UN~[1-5000]
Nbot12	N	UN~[1-10]
Nbot22	N	UN~[1-10]
Nbot32	N	UN~[1-10]
Nanc2	N	UN~[10-20000]
Texp2	T	UN~[10-5000]
db2	T	UN~[1-10]
N13 (<Nexp3)	N	UN~[1-5000]
N23 (<Nexp3)	N	UN~[1-5000]
N33 (<Nexp3)	N	UN~[1-5000]
Nexp3	N	UN~[11-10000]
Nbot3 (<N13, N23, N33)	N	UN~[1-10]
Nanc3	N	UN~[10-20000]
Tisol3 (<Texp3)	T	UN~[10-5000]
Texp3	T	UN~[10-5000]
db3	T	UN~[1-10]
N14	N	UN~[1-100]
N24	N	UN~[1-100]
N34	N	UN~[1-100]
Niso14 (<Nexp4)	N	UN~[101-5000]
Niso24 (<Nexp4)	N	UN~[101-5000]
Niso34 (<Nexp4)	N	UN~[101-5000]
Nexp4	N	UN~[101-10000]
Nbot4	N	UN~[1-10]
Nanc4	N	UN~[10-20000]
Tcrash14	T	UN~[1-50]
Tcrash24	T	UN~[1-50]
Tcrash34	T	UN~[1-50]
Tisol4 (<Texp4)	T	UN~[51-5000]
Texp4	T	UN~[51-5000]
db4	T	UN~[1-10]
N15 (<Nexp5)	N	UN~[1-5000]
N25 (<Nexp5)	N	UN~[1-5000]
N35 (<Nexp5)	N	UN~[1-5000]
Nexp5 (>Nfond5)	N	UN~[10-10000]
Nfond5	N	UN~[1-10]
Tisol5 (<Texp5)	T	UN~[10-5000]
Texp5	T	UN~[10-5000]
N16 (<Nexp6)	N	UN~[1-5000]
N26 (<Nexp6)	N	UN~[1-5000]
N36 (<Nexp6)	N	UN~[1-5000]
Nexp6	N	UN~[10-10000]
Nanc6	N	UN~[10-5000]
Tisol6 (<Texp6)	T	UN~[10-5000]
Texp6	T	UN~[10-5000]

Table S2 (*Continue)*

Step b_{ii} in Fig. 4

**Demographic Parameter**	**Type**	**Prior**
N11(>Nbot1)	N	UN~[1, 5000]
N21(>Nbot1)	N	UN~[1, 5000]
N31(>Nbot1)	N	UN~[1, 5000]
Nexp1(>N11, N12,N13)	N	UN~[11, 10000]
Nbot1	N	UN~[1, 10]
Nanc1	N	UN~[10, 20000]
Tisol1(<Texp1)	T	UN~[10, 5000]
Texp1	T	UN~[10, 5000]
db1	T	UN~[1, 10]
N12	N	UN~[1, 100]
N22	N	UN~[1, 100]
N32	N	UN~[1, 100]
Niso12(>N12)	N	UN~[1, 5000]
Niso22(>N22)	N	UN~[1, 5000]
Niso23(>N32)	N	UN~[1, 5000]
Nexp2	N	UN~[11, 10000]
Nbot2	N	UN~[1, 10]
Nanc2	N	UN~[10, 20000]
Tcrash12	T	UN~[1, 5]
Tcrash22	T	UN~[1, 5]
Tcrash23	T	UN~[1, 5]
Tisol2(>Tcrash12, Tcrash22, Tcrash23)	T	UN~[10, 5000]
Texp2(>Tisol2)	T	UN~[10, 5000]
db2	T	UN~[1, 10]

Table S3. Mutation model parameters for the ABC analysis (see figure 4)

**Microsatellites mutation parameter**	**GSM (40 steps allowed)**
$\overline{\mu}_{mic}$	UN~[1 x 10^{-4} – 1 x10^{-3}]
$G_{\mu mic}$	GA~[1 x 10^{-5}, 1 x 10^{-2}, $\overline{\mu}_{mic}$, 2]
$\overline{P}$	UN~[1 x 10^{-1}, 3 x 10^{-1}]
GP	GA~[1 x10^{-2}, 9 x 10^{-1}, $\overline{P}$, 2]
SNI	LU~[1 x10^{-8}, 1 x10^{-5}]
G_{SNI}	GA~[1 x10^{-9}, 1 x10^{-4}, SNI, 2]
**MtDNA mutation parameter**	HKY (*p-inv*: 15.6, α: 99.8)
μ_{seq}	UN~[1 x 10^{-7}, 1 x 10^{-5}]
K1	UN~[0.050, 20]

The mutation model parameters for the microsatellite loci were the mean mutation rate (μ_{mic}), the parameter determining the shape of the gamma distribution of individual loci mutation rate (*P*), and the Single Insertion Nucleotide rate (*SNI*). The mtDNA mutation model was is an HKY with two variable parameters, the per-site and generation mutation rate (μ_{seq}) and the transition/transversion ratio (*K1*) parameter, and two fixed parameters, the proportion of constant sites (*p-inv.*), and the shape of the Gamma distribution of mutations among sites (α).

Table S4. Model selection procedure and performance analysis for the ABC step (*a*) in figure 4*a*.

ABC-RF – Posterior probability ± s.d. (Prior Error rate ± s.d.)

	SC1	SC2	SC3	SC4	SC5	SC6	SC7	SC8	SC9	SC10
	**55.8 ± 2.5%<br>(62.9 ± 0.1%)**	--	--	--	--	--	--	--	--	--

ABC-RF classification votes (based on 500 decision trees, a reference table of $1\text{x}10^4$ PODs per scenario)

	SC1	SC2	SC3	SC4	SC5	SC6	SC7	SC8	SC9	SC10
Mean±sd (10 rep.)	**181.0 ± 12.0**	8.5 ± 2.7	23.8 ±4.9	44.0 ± 6.8	85.4 ± 11.4	49.6 ± 6.8	31.9 ± 5.1	33.7 ± 5.7	26.1 ± 5.0	16.0 ± 4.5
Mean±sd (%)	**36.2±2.4**	1.7±0.5	4.8±1.0	8.8±1.4	17.1±2.3	9.9±1.4	6.4±1.1	6.7±1.1	5.2±1.0	3.2±0.9

“Standard” ABC-LDA: Posterior probability [95 CI]

	SC1	SC2	SC3	SC4	SC5	SC6	SC7	SC8	SC9	SC10
	**66.7%**	1.3%	1.4%	7.7%	5.8%	7.1%	2.8%	3.3%	2.4%	1.5%
	**[65.6-67.8]**	[1.2 - 1.5]	[1.2 – 1.6]	[7.1 – 8.2]	[5.3 – 6.2]	[6.6 – 7.7]	[2.5 – 3.0]	[3.0 – 3.6]	[2.1 – 2.6]	[1.3 – 1.7]

Performance–ABC-LDA (based on 300 simulated data set and a reference table of 1×10^6 PODs per scenario)

	SC1	SC2	SC3	SC4	SC5	SC6	SC7	SC8	SC9	SC10
**D1**	**61.4%**	***11.6%†***	***18.3%†***	***14.3%†***	***13.7%†***	***12.0%†***	***9.0%†***	***14.0%†***	***11.8%†***	***11.7%†***
**D2**	*1.7%**	38.5%	17.6%	0.7%	1.8%	1.0%	0.3%	0.7%	0.0%	18.7%
**D3**	*7.6%**	21.1%	30.0%	3.0%	2.4%	4.0%	3.0%	6.0%	3.0%	17.7%
**D4**	*3.0%**	1.0%	1.3%	40.3%	24.0%	20.0%	1.0%	1.0%	1.6%	1.0%
**D5**	*9.7%**	2.3%	4.3%	18.6%	33.4%	22.3%	4.3%	5.3%	6.0%	5.3%
**D6**	*2.3%**	1.6%	1.0%	18.0%	17.1%	35.3%	1.0%	0.7%	0.7%	0.3%
**D7**	*3.0%**	0.3%	1.0%	1.7%	1.0%	1.7%	34.0%	17.3%	23.3%	0.3%
**D8**	*7.0%**	1.7%	4.7%	3.0%	5.5%	3.3%	23.0%	34.0%	18.0%	2.7%
**D9**	*2.3%**	0.0%	0.7%	0.0%	0.8%	0.3%	23.0%	20.7%	35.3%	0.3%
**D10**	*2.0%**	22.0%	21.0%	0.3%	0.3%	0.0%	1.3%	0.3%	0.3%	42.0%

Goodness of fit of the fitted model to the data (number of outlying statistics, see table S6)

**$P < 0.05$**	1	3	7	9	4	8	2	4	4	1
**$P < 0.01$**	4	1	2	1	0	2	2	0	0	3
**$P < 0.001$**	**0**	0	0	0	0	0	0	0	0	1

D: proportion of case in which the simulation-based ABC-LDA model choice procedure was able to select a scenario as the most probable with non-overlapping confidence intervals of the posterior probabilities of each scenario.
* Type-I or α-error rate; † Type-II or β-error rate and that 1- Σ βi provide the power of the model choice procedure.
Prior error rates, posterior probabilities, and classification votes of the best model chosen using ABC-RF were averaged over 10 replicated ABC-RF analyses.

Table S5. Model checking for the first step (see figure 4*a*).

	OBS. VALUE	SC1	SC2	SC3	SC4	SC5	SC6	SC7	SC8	SC9	SC10
MGW_1_1	0.5121	0.0055 (**)	0.0130 (*)	0.0105 (*)	0.0120 (*)	0.0115 (*)	0.0100 (**)	0.0090 (**)	0.0220 (*)	0.0115 (*)	0.0065 (**)
MGW_1_2	0.4598	0.0065 (**)	0.0175 (*)	0.01 (**)	0.0175 (*)	0.0160 (*)	0.0220 (*)	0.0110 (*)	0.0175 (*)	0.0130 (*)	0.0165 (*)
MGW_1_3	0.4403	0.006 (**)	0.0115 (*)	0.01 (**)	0.0100 (**)	0.0125 (*)	0.0060 (**)	0.0035 (**)	0.0150 (*)	0.0200 (*)	0.0060 (**)
DM2_1_1&2	5.0991	0.955 (*)	0.87	0.9095	0.9610 (*)	0.9570 (*)	0.9720 (*)	0.888	0.866	0.872	0.9115
NHA_2_3	1	0.053	0.0605	0.0475 (*)	0.0455 (*)	0.059	0.0485 (*)	0.0755	0.066	0.0825	0.0665
NSS_2_3	0	0.053	0.0605	0.0475 (*)	0.0455 (*)	0.059	0.0485 (*)	0.0755	0.066	0.0825	0.0665
MPD_2_3	0	0.053	0.0605	0.0475 (*)	0.0455 (*)	0.059	0.0485 (*)	0.0755	0.066	0.0825	0.0665
VPD_2_3	0	0.053	0.0605	0.0475 (*)	0.0455 (*)	0.059	0.0485 (*)	0.0755	0.066	0.0825	0.0665
MNS_2_3	0	0.053	0.0605	0.0475 (*)	0.0455 (*)	0.059	0.0485 (*)	0.0755	0.066	0.0825	0.0665
HST_2_2&3	0.7656	0.995 (**)	0.9950 (**)	0.986 (*)	0.9740 (*)	0.9565 (*)	0.9530 (*)	0.9570 (*)	0.9790 (*)	0.9640 (*)	0.9940 (**)

Scenarios are shown in figure 4. The probability *Prob.* ($S_{simul.} < S_{obs.}$) for each summary statistic is calculated from 1,000 PODs simulated from the posterior distributions of parameters obtained under the each focal scenario. Corresponding tail-area probabilities (*p*-values) were obtained as *Prob.* ($S_{simul.} < S_{obs.}$) and 1.0 - *Prob.* ($S_{simul.} < S_{obs.}$) for *Prob*. ($S_{simul.} < S_{obs.}$) ≤ 0.5 and > 0.5, respectively (*, **, *** = tail-area probability < 0.05, < 0.01 and < 0.001, respectively). In addition to the statistics used during the model choice procedure, the model check procedure used also two samples statistics including the mean number of alleles, mean genetic diversity, and mean size variance for microsatellite loci; and for mtDNA the within sample statistics including mean number of the rarest nucleotide at segregating sites and its variance, and the two samples statistics comprising the number of haplotypes and number of segregating sites. Only significant summary statistics for at least one scenario are shown. Abbreviations for the summary statistics are as follows: Mean Garcia-Williamson index (MGW), $d\mu^2$ distance (DM2); Number of mtDNA haplotypes (NHA); Number of segregating site (NSS); Mean pairwise difference (MPD); Variance of pariwise difference (VPD); Mean number of the rarest nucleotide at segregating sites (MSN), Hudson's FST-statistics (HST)

Table S6. Model selection procedure and performance analysis for the ABC step (*bi*), figure 4*b*.

**ABC-RF – Posterior probability ± s.d. (Prior Error rate)**						
	SC1	SC2	**SC3**	SC4	SC5	SC6
	--	--	**69.8 ± 1.9% (41.4% ± 0.1%)**	--	--	--
**ABC-RF – Posterior probability ± s.d. (Prior Error rate)**						
Mean ± sd (10 rep., count)	39.4±6.5	21.1±6.6	**335.2±13.9**	18.6±5.6	76.7±9.2	9.0±2.9
Mean ± sd (%)	7.9±1.3%	4.2±1.3%	**67.0±2.8%**	3.7±1.1%	15.3±1.8%	1.8±0.6%
**“Standard” ABC-LDA: Posterior probability [95 CI]**						
**Post.Pr**	6.10%	3.40%	**77.80%**	4.50%	7.20%	0.10%
**95%CI**	[5.6 – 6.7]	[3.0 – 3.9]	**[76.8 – 78.9]**	[4.0 – 4.9]	[6.7 - 7.7]	[0.0 – 0.1]
**Performance–ABC-LDA** (based on 300 simulated data set and a reference table of 1 x 10^6 PODs per scenario)						
	**SC1**	**SC2**	**SC3**	**SC4**	**SC5**	**SC6**
**D1**	47.30%	17.30%	***13.70%****	6.70%	7.30%	0.00%
**D2**	14.00%	60.00%	***7.00%****	2.33%	4.70%	0.00%
**D3**	***11.30%†***	***5.30%†***	***37.30%***	***6.30%†***	***13%†***	***0.30%†***
**D4**	8.70%	9.30%	***20.30%****	78.70%	34.00%	0.00%
**D5**	18.30%	6.30%	***20.70%****	12.00%	41.00%	0.00%
**D6**	0.30%	1.70%	***1.00%****	0.00%	0.00%	99.70%
**Goodness of fit of the fitted model to the data** (number of outlying statistics, see table S8)						
***P* < 0.05**	8	4	**0**	9	1	7
***P* < 0.01**	2	1	**1**	0	4	0
***P* < 0.001**	0	0	**0**	3	0	0

(see the legend of table S4)

Table S7. Model checking for the ABC step *bi* (figure 4*bi*).

	Obs. value	SC1	SC2	SC3	SC4	SC5	SC6
VAR_1_1	11.2626	0.686	0.68	**0.43**	0.9600 (*)	0.85	0.644
VAR_1_2	16.2651	0.833	0.836	**0.618**	0.9850 (*)	0.937	0.834
VAR_1_3	15.9953	0.837	0.823	**0.627**	0.9870 (*)	0.928	0.843
MGW_1_1	0.5121	0.0070 (**)	0.0130 (*)	**0.0975**	0.0000 (***)	0.0040 (**)	0.0350 (*)
MGW_1_2	0.4598	0.0135 (*)	0.0190 (*)	**0.12**	0.0010 (***)	0.0030 (**)	0.0365 (*)
MGW_1_3	0.4403	0.0115 (*)	0.0090 (**)	**0.136**	0.0010 (***)	0.0050 (**)	0.0375 (*)
V2P_1_1&2	13.0832	0.738	0.74	**0.489**	0.9710 (*)	0.882	0.725
V2P_1_1&3	12.4376	0.719	0.722	**0.469**	0.9660 (*)	0.873	0.683
V2P_1_2&3	16.8347	0.847	0.829	**0.616**	0.9870 (*)	0.935	0.8355
FST_1_1&2	0.0484	0.7255	0.7345	**0.573**	0.3995	0.597	0.0425 (*)
FST_1_2&3	0.0533	0.667	0.737	**0.559**	0.3885	0.591	0.0475 (*)
LIK_1_1&2	1.2477	0.242	0.338	**0.241**	0.3765	0.365	0.0480 (*)
DM2_1_1&2	5.0991	0.9620 (*)	0.9570 (*)	**0.898**	0.9750 (*)	0.9790 (*)	0.913
DM2_1_2&3	3.638	0.904	0.895	**0.739**	0.9580 (*)	0.942	0.754
NHA_2_3	1	0.0475 (*)	0.064	**0.088**	0.055	0.057	0.1035
NSS_2_3	0	0.0475 (*)	0.064	**0.088**	0.055	0.057	0.1035
MPD_2_3	0	0.0475 (*)	0.064	**0.088**	0.055	0.057	0.1035
VPD_2_3	0	0.0475 (*)	0.064	**0.088**	0.055	0.057	0.1035
MNS_2_3	0	0.0475 (*)	0.064	**0.088**	0.055	0.057	0.1035
HST_2_2&3	0.7656	0.9920 (**)	0.9790 (*)	**0.9900 (**)**	0.9820 (*)	0.9940 (**)	0.9660 (*)

The probability *Prob*.($S_{simul.} < S_{obs.}$) given for each summary statistic was calculated from 1,000 virtual datasets simulated from the posterior distributions of parameters obtained under the focused scenario. Corresponding tail-area probabilities (*p*-values) were obtained as *Prob*. ($S_{simul.} < S_{obs.}$) and 1.0 - *Prob*. ($S_{simul.} < S_{obs.}$) for *Prob*. ($S_{simul.} < S_{obs.}$) ≤ 0.5 and > 0.5, respectively (*, **, *** = tail-area probability < 0.05, < 0.01 and < 0.001, respectively). In addition to the statistics used during the model choice procedure, the model check procedure used also two samples statistics including the mean number of alleles, mean genetic diversity, and mean size variance for microsatellite loci; and for mtDNA the within sample statistics including mean number of the rarest nucleotide at segregating sites and its variance, and the two samples statistics comprising the number of haplotypes and number of segregating sites. Only significant summary statistics for at least one scenario are shown. Abbreviations for the summary statistics are as follows: Mean size variance (VAR); Mean Garcia-Williamson index (MGW); Variance of pairwise difference (V2P); F_{ST} between 2 samples (FST); $d\mu^2$ distance (DM2); Classification index (LIK); Number of mtDNA haplotypes (NHA); Number of segregating sites (NSS); Mean pairwise difference (MPD); Variance of pairwise difference (VPD); Mean number of the rarest nucleotide at segregating sites (MSN); Hudson's FST-statistics (HST).

Table S8. Model selection procedure and performance analysis for the ABC step (*bii*), figure 4*b*.

**ABC-RF – Posterior probability ± s.d. (Prior Error rate)**		
	SC1	**SC2**
	--	**69.2 ± 2.0% (15.9 ± 0.1%)**
**ABC-RF classification votes** (based on 500 decision trees, a reference table of 1x10^4 PODs per scenario)		
Mean±sd (10 rep., count)	176.7±9.9	**323.3±9.9**
Mean±sd (%)	35.3±2.0%	**64.7±2.0%**
**ABC-LDA – Posterior probability [95 CI]**		
Post. Pr	21.8%	**78.2%**
95%CI	[21.1 – 22.5]	**[77.5 – 78.9]**
**Performance–ABC-LDA** (based on 300 simulated data set and a reference table of 1 x 10^6 PODs per scenario)		
**Decision**	**SC1**	**SC2**
**D1**	86.50%	**14.50%**
**D2**	13.50%	**85.50%**
**Goodness of fit of the fitted model to the data** (number of outlying statistics, see table S10)		
$P < 0.05$	0	**0**
$P < 0.01$	1	**1**
$P < 0.001$	0	**0**

(see legend of table S4)

Table S9. Model checking for the analysis for the ABC step (*bii*), figure 4*b*.

	Obs. value	SC1	SC2
HST_2_2&3	0.7656	0.9950(**)	0.9950(**)

Hudson's FST-statistics (HST)

Table S10. Parameter estimation for scenario SC2 in natural units (Fig. 4b_{ii}).

	mean	mode	$Q_{2.5}$	$Q_{5.0}$	$Q_{25.0}$	median	$Q_{75.0}$	$Q_{95.0}$	$Q_{97.5}$
$N_{12\text{–}PY}$	49.6	**35.3**	11.3	14.6	29.7	46.5	68.3	92.7	96.0
$N_{22\text{–}TL}$	54.8	**32.3**	11.4	15.5	32.9	53.8	77.2	95.8	97.9
$N_{32\text{–}XCSS}$	42.3	**13.9**	6.9	8.8	20.0	36.3	62.5	91.0	95.4
N_{iso12}	2,520.0	**2,090.0**	599.0	772.0	1,580.0	2,380.0	3,410.0	4,620.0	4,810.0
N_{iso22}	2,450.0	**1,720.0**	591.0	756.0	1,530.0	2,300.0	3,300.0	4,600.0	4,790.0
N_{iso32}	2,420.0	**2,030.0**	578.0	758.0	1,470.0	2,250.0	3,280.0	4,600.0	4,800.0
N_{exp2}	6,580.0	**5,660.0**	2,900.0	3,360.0	5,090.0	6,570.0	8,170.0	9,620.0	9,840.0
N_{bot2}	6.8	**10.0**	1.8	2.6	5.1	7.1	8.7	10.0	10.0
N_{anc2}	13,300.0	**18,700.0**	3,480.0	4,830.0	10,100.0	13,900.0	17,200.0	19,500.0	19,700.0
$T_{crash12}$	3.0	**1.0**	1.0	1.0	1.5	2.9	4.5	5.0	5.0
$T_{crash22}$	2.6	**1.0**	1.0	1.0	1.2	2.2	3.7	5.0	5.0
$T_{crash32}$	3.2	**5.0**	1.0	1.0	2.1	3.4	4.6	5.0	5.0
T_{isol2}	154.0	**103.0**	21.4	31.4	76.7	126.0	200.0	365.0	448.0
T_{exp2}	1,060.0	**340.0**	130.0	178.0	394.0	682.0	1,320.0	3,410.0	4,100.0
db	6.1	**6.4**	1.1	1.9	4.1	6.2	8.1	10.0	10.0
μ_{mic_1}	5.81E-04	**4.48E-04**	2.13E-04	2.48E-04	4.11E-04	5.68E-04	7.48E-04	9.43E-04	9.76E-04
p_{mic_1}	2.48E-01	**3.00E-01**	1.26E-01	1.43E-01	2.19E-01	2.62E-01	2.89E-01	3.00E-01	3.00E-01
sni_{mic_1}	1.05E-06	**1.00E-08**	1.13E-08	1.31E-08	4.49E-08	2.16E-07	1.07E-06	5.54E-06	7.33E-06
u_{seq_2}	9.96E-07	**5.61E-07**	2.33E-07	2.94E-07	5.41E-07	8.16E-07	1.23E-06	2.27E-06	2.86E-06
$k1_{seq\_2}$	1.02E+01	**1.69E+01**	5.52E-01	1.03E+00	5.10E+00	1.03E+01	1.52E+01	1.90E+01	1.95E+01

Evolutionary scenario SC2 are represented in Fig. 4*bii*.; Effective population size is provided in term of diploid individuals; Time of the event are provided in generation before present. Q_x: *x* % quantile.

Table S11. Composite parameter for the scenario SC2 (see Fig. 4*bii*)

Parameter	mean	mode	$Q_{2.5}$	$Q_{5.0}$	$Q_{25.0}$	median	$Q_{75.0}$	$Q_{95.0}$	$Q_{97.5}$
N12(u+sni)_1	2.48E-02	**1.36E-02**	3.96E-03	5.41E-03	1.23E-02	2.05E-02	3.28E-02	5.95E-02	6.98E-02
N22(u+sni)_1	3.12E-02	**1.16E-03**	9.00E-04	1.46E-03	7.78E-03	2.23E-02	4.91E-02	8.86E-02	9.51E-02
N32(u+sni)_1	2.49E-02	**1.09E-02**	3.22E-03	4.56E-03	1.11E-02	1.99E-02	3.40E-02	6.34E-02	7.30E-02
Niso12(u+sni)_1	2.87E+00	**4.98E+00**	2.99E-01	4.84E-01	1.71E+00	3.00E+00	4.09E+00	4.94E+00	4.98E+00
Niso22(u+sni)_1	1.49E+00	**1.05E+00**	3.27E-01	4.27E-01	8.34E-01	1.26E+00	1.93E+00	3.43E+00	3.90E+00
Niso32(u+sni)_1	1.15E+00	**3.82E-01**	1.22E-01	1.69E-01	4.39E-01	8.26E-01	1.54E+00	3.30E+00	3.96E+00
Nexp2(u+sni)_1	4.88E+00	**3.37E+00**	3.82E-01	6.40E-01	2.55E+00	4.71E+00	7.20E+00	9.58E+00	9.98E+00
Nbot2(u+sni)_1	3.73E-03	**2.99E-03**	9.11E-04	1.12E-03	2.32E-03	3.47E-03	4.91E-03	7.23E-03	7.96E-03
Nanc2(u+sni)_1	4.32E+00	**9.95E-01**	2.54E-01	3.96E-01	1.41E+00	3.08E+00	5.99E+00	1.27E+01	1.51E+01
Tcrash12(u+sni)_1	1.28E-03	**6.16E-04**	2.19E-04	2.68E-04	5.79E-04	1.01E-03	1.72E-03	3.29E-03	3.82E-03
Tcrash22(u+sni)_1	2.14E-03	**1.07E-04**	1.38E-04	2.08E-04	9.14E-04	1.96E-03	3.23E-03	4.68E-03	4.94E-03
Tcrash32(u+sni)_1	1.29E-03	**1.09E-04**	1.14E-04	1.25E-04	2.53E-04	6.67E-04	1.88E-03	4.50E-03	4.93E-03
Tisol2(u+sni)_1	8.39E-02	**3.21E-02**	1.11E-02	1.49E-02	3.61E-02	6.20E-02	1.05E-01	2.18E-01	2.84E-01
Texp2(u+sni)_1	1.92E-01	**3.05E-02**	1.99E-02	2.28E-02	4.45E-02	8.66E-02	1.98E-01	6.94E-01	1.04E+00
db(u+sni)_1	3.87E-03	**1.49E-03**	5.99E-04	8.27E-04	2.03E-03	3.51E-03	5.43E-03	7.99E-03	8.63E-03
N12useq_2	4.92E-05	**2.32E-05**	5.63E-06	8.53E-06	2.15E-05	3.72E-05	6.18E-05	1.28E-04	1.66E-04
N22useq_2	5.58E-05	**2.39E-06**	1.24E-06	1.95E-06	7.34E-06	2.01E-05	5.53E-05	2.42E-04	3.69E-04
N32useq_2	2.49E-05	**7.08E-07**	5.47E-07	7.63E-07	2.99E-06	8.41E-06	2.33E-05	1.00E-04	1.64E-04
Niso12useq_2	2.01E-03	**9.26E-04**	3.75E-04	4.82E-04	9.97E-04	1.57E-03	2.45E-03	4.92E-03	6.25E-03
Niso22useq_2	2.42E-03	**1.06E-03**	3.29E-04	4.38E-04	9.91E-04	1.69E-03	2.85E-03	6.69E-03	9.39E-03
Niso32useq_2	3.96E-03	**1.97E-04**	8.98E-05	1.45E-04	6.19E-04	1.67E-03	4.47E-03	1.68E-02	2.34E-02
Nexp2useq_2	9.61E-03	**9.26E-04**	4.98E-04	6.61E-04	1.84E-03	4.27E-03	1.06E-02	4.01E-02	5.57E-02
Nbot2useq_2	4.77E-05	**1.98E-07**	1.24E-06	2.53E-06	1.79E-05	4.64E-05	7.65E-05	9.80E-05	9.83E-05
Nanc2useq_2	3.52E-02	**1.67E-02**	2.84E-03	4.34E-03	1.36E-02	2.65E-02	4.82E-02	9.75E-02	1.16E-01
Tcrash12useq_2	5.67E-06	**1.49E-06**	6.54E-07	9.02E-07	2.31E-06	4.27E-06	7.48E-06	1.52E-05	1.90E-05
Tcrash22useq_2	2.63E-06	**1.28E-06**	3.89E-07	5.05E-07	1.15E-06	1.98E-06	3.30E-06	6.80E-06	8.71E-06
Tcrash32useq_2	3.33E-06	**1.06E-06**	4.65E-07	6.09E-07	1.43E-06	2.54E-06	4.28E-06	8.69E-06	1.08E-05
Tisol2useq_2	1.28E-04	**4.49E-05**	1.47E-05	2.12E-05	5.28E-05	9.44E-05	1.63E-04	3.43E-04	4.32E-04
Texp2useq_2	3.15E-03	**6.41E-05**	5.07E-05	8.23E-05	3.87E-04	1.14E-03	3.29E-03	1.37E-02	1.99E-02
dbuseq_2	7.99E-06	**2.42E-07**	2.02E-07	2.93E-07	1.27E-06	3.57E-06	9.78E-06	3.10E-05	4.29E-05

References:

1. Estoup, A., Jarne, P. & Cornuet, J.-M. 2002 Homoplasy and mutation model at microsatellite loci and their consequences for population genetics analysis. *Mol. Ecol.* **11**, 1591–1604.

2. Darriba, D., Taboada, G. L., Doallo, R. & Posada, D. 2012 jModelTest 2: more models, new heuristics and parallel computing. *Nat Meth.* **9**, 772. (doi:10.1038/nmeth.2109)

3. Hasegawa, M., Kishino, H. & Yano, T. 1985 Dating of the human-ape splitting by a molecular clock of mitochondrial DNA. *J. Mol. Evol.* **22**, 160–174.

4. Alter, S. E. & Palumbi, S. R. 2009 Comparing evolutionary patterns and variability in the mitochondrial control region and cytochrome B in three species of baleen whales. *J. Mol. Evol.* **68**, 97–111. (doi:10.1007/s00239-008-9193-2)

5. Fontaine, M. C. et al. 2010 Genetic and historic evidence for climate-driven population fragmentation in a top cetacean predator: the harbour porpoises in European water. *Proc. R. Soc. B* **277**, 2829–2837. (doi:10.1098/rspb.2010.0412)

6. Garza, J. C. & Williamson, E. G. 2001 Detection of reduction in population size using data from microsatellite loci. *Mol. Ecol.* **10**, 305–318. (doi:10.1046/j.1365-294x.2001.01190.x)

7. Weir, B. S. & Cockerham, C. C. 1984 Estimating F-statistics for the analysis of population structure. *Evolution*, 1358–1370.

8. Chakraborty, R. & Jin, L. 1993 A unified approach to study hypervariable polymorphisms: statistical considerations of determining relatedness and population distances. *EXS* **67**, 153–175.

9. Goldstein, D. B., Ruiz Linares, A., Cavalli-Sforza, L. L. & Feldman, M. W. 1995 An evaluation of genetic distances for use with microsatellite loci. *Genetics* **139**, 463–471.

10. Hudson, R. R., Slatkin, M. & Maddison, W. P. 1992 Estimation of levels of gene flow from DNA sequence data. *Genetics* **132**, 583–589.

11. Beaumont, M. A., Zhang, W. & Balding, D. J. 2002 Approximate Bayesian computation in population genetics. *Genetics* **162**, 2025–2035.

12. Cornuet, J.-M., Santos, F., Beaumont, M. A., Robert, C. P., Marin, J.-M., Balding, D. J., Guillemaud, T. & Estoup, A. 2008 Inferring population history with DIY ABC: a user-friendly approach to approximate Bayesian computation. *Bioinformatics* **24**, 2713–2719. (doi:10.1093/bioinformatics/btn514)

13. Cornuet, J.-M., Ravignie, V. & Estoup, A. 2010 Inference on population history and model checking using DNA sequence and microsatellite data with the software DIYABC (v1.0). *BMC Bioinfor.* **11**, 401. (doi:10.1186/1471-2105-11-401)

14. Estoup, A., Lombaert, E., Marin, J.-M., Guillemaud, T., Pudlo, P., Robert, C. P. & Cornuet, J.-M. 2012 Estimation of demo-genetic model probabilities with Approximate Bayesian Computation using linear discriminant analysis on summary statistics. *Mol. Ecol. Resour.* **12**, 846–855. (doi:10.1111/j.1755-0998.2012.03153.x)

15. Pudlo, P., Marin, J.-M., Estoup, A., Cornuet, J.-M., Gautier, M. & Robert, C. P. 2016 Reliable ABC model choice via random forests. *Bioinformatics* **32**, 859–866. (doi:10.1093/bioinformatics/btv684)

16. Blum, M. G. B., Nunes, M. A., Prangle, D. & Sisson, S. A. 2013 A Comparative Review of Dimension Reduction Methods in Approximate Bayesian Computation. *Statistical Science* **28**, 189–208. (doi:10.2307/43288487?ref=search-gateway:5071db5de9fc3b9e6fd3c6c3a7505178)

17. Robert, C. P., Cornuet, J.-M., Marin, J.-M. & Pillai, N. S. 2011 Lack of confidence in approximate Bayesian computation model choice. *P. Natl. Acad. Sci. USA* **108**, 15112–15117. (doi:10.1073/pnas.1102900108)

18. Fraimout, A. et al. 2017 Deciphering the routes of invasion of Drosophila suzukii by means of ABC random forest. *Mol. Biol. Evol.* (doi:10.1093/molbev/msx050)

19. Rannala, B. & Mountain, J. L. 1997 Detecting immigration by using multilocus genotypes. *P. Natl. Acad. Sci. USA* **94**, 9197–9201.

20. Pascual, M., Chapuis, M.-P., Mestres, F., Balanyà, J., Huey, R. B., Gilchrist, G. W., Serra, L. & Estoup, A. 2007

Introduction history of Drosophila subobscura in the New World: a microsatellite-based survey using ABC methods. *Mol. Ecol.* **16**, 3069–3083. (doi:10.1111/j.1365-294X.2007.03336.x)

21. Gelman, A., Carlin, J. B., Stern, H. S., Dunson, D. B., Vehtari, A. & Rubin, D. B. 2004 *Bayesian Data Analysis, Second Edition*. CRC Press.

22. Szpiech, Z. A., Jakobsson, M. & Rosenberg, N. A. 2008 ADZE: a rarefaction approach for counting alleles private to combinations of populations. *Bioinformatics* **24**, 2498–2504. (doi:10.1093/bioinformatics/btn478)

23. Do, C., Waples, R. S., Peel, D., Macbeth, G. M., Tillett, B. J. & Ovenden, J. R. 2014 NeEstimator v2: re-implementation of software for the estimation of contemporary effective population size (*Ne*) from genetic data. *Mol. Ecol. Resour.* **14**, 209–214. (doi:10.1111/1755-0998.12157)

24. Tallmon, D. A., Koyuk, A., Luikart, G. & Beaumont, M. A. 2008 ONESAMP: a program to estimate effective population size using approximate Bayesian computation. *Mol. Ecol. Resour.* **8**, 299–301. (doi:10.1111/j.1471-8286.2007.01997.x)
